# Molecular Characterization of MyD88 in *Anodonta woodiana* and Its Involvement in the Innate Immune Response to Bacterial Infection

**DOI:** 10.3389/fimmu.2022.925168

**Published:** 2022-06-10

**Authors:** Fufa Qu, Qing She, Jialing Li, Xuan Zeng, Yumiao Li, Xinyu Liu, Lingxin Ren, Zhenzhen Liu, Chaoran Gao, Xinyu Lu, Mengyao Long, Xinya Li

**Affiliations:** Hunan Provincial Key Laboratory of Nutrition and Quality Control of Aquatic Animals, Department of Biological and Environmental Engineering, Changsha University, Changsha, China

**Keywords:** *Anodonta woodiana*, MyD88, signaling pathway, bacterial challenge, innate immunity

## Abstract

Myeloid differentiation factor 88 (MyD88) is a key adapter molecule in Toll-like receptor signal transduction that triggers downstream immune cascades involved in the host defense response to exogenous pathogens. However, the function of MyD88s in mollusks, especially in freshwater shellfish, remains poorly understood. In this study, a novel freshwater shellfish MyD88 (denoted *Aw*MyD88) was characterized from *Anodonta woodiana*. The present *Aw*MyD88 protein consists of 474 amino acids and contains a conserved a typical death domain (DD) and a conservative Toll/IL-1R (TIR) domain with three typical boxes. Quantitative real-time PCR (qRT-PCR) analysis showed that *Aw*MyD88 was broadly expressed in all the examined tissues, and the highest expression level was observed in hemocytes of *A. woodiana*. When challenged with *Aeromonas hydrophila* and lipopolysaccharide (LPS), the mRNA expression levels of *Aw*MyD88 were significantly induced in hemocytes of *A. woodiana in vivo* and *in vitro*. In addition, *in vivo* injection experiments revealed that MyD88 signaling pathway genes showed strong responsiveness to *A. hydrophila* challenge, and their expression levels were significantly upregulated in hemocytes. Knockdown of *Aw*MyD88 reduced the transcript levels of immune related transcription factors (*Aw*NF-κB and *Aw*AP-1) and effectors (*Aw*TNF, *Aw*LYZ, *Aw*Defense and *Aw*AIF) during *A. hydrophila* infection. Moreover, subcellular localization analysis indicated that *Aw*MyD88 was mainly localized to the cytoplasm in HEK293T cells. Finally, luciferase reporter assays revealed that *Aw*MyD88 associates with *Aw*TLR to activate the NF-κB and AP-1 signaling pathways in HEK293T cells. These results suggested that *Aw*MyD88 might be involved in the host defense response to bacterial challenge, providing new insight into the immune function of the MyD88 signaling pathway in freshwater shellfish.

## Introduction

Innate immunity is the first line of defense against pathogen infections and plays a vital role in the host immune system (1, 2). The innate immune system detects exogenous threats or endogenous stress mainly through pattern recognition receptors (PRRs), which are responsible for sensing the conserved molecular structures of pathogens, termed pathogen-associated molecular patterns (PAMPs) (3, 4). Toll-like receptors (TLRs) are the most important classes of evolutionarily conserved innate PRRs that play a crucial role in host defense by recognizing conserved PAMPs such as lipopolysaccharide (LPS), peptidoglycan (PGN) and flagellin (5, 6). Upon immune stimulation, TLRs are activated and then recruit the cytosolic adaptor protein myeloid differentiation factor 88 (MyD88) through TIR-TIR domain interactions. The death domain of MyD88 subsequently associates with the death domain of IL-1R-associated kinases (IRAKs). IRAKs dissociate with MyD88, form a complex with TRAF6, then phosphorylate TGF‐β‐activated kinase‐1 (TAK1) and TAK1-binding protein (TAB) complex and induce the activation of the transcription factors nuclear factor-kappa B (NF-κB) and activator protein-1 (AP-1). This process ultimately results in the production of inflammatory cytokines, antimicrobial peptides, chemokines, adhesion molecules and lysozymes, thus initiating the innate immune response (6–8).

MyD88 is an essential adaptor molecule in the TLR signaling pathway [6] and can be recruited by all TLRs except TLR3 in vertebrate (9, 10). It was shown that MyD88 could interact directly with the C-terminal Toll-like/IL-1 receptor (TIR) domain of TLRs and the death domain (DD) of IRAKs, which is essential for triggering the immune defense response during pathogen challenges (11–13). In mammals, Kawai and coworkers showed that treatment with the TLR4 ligand LPS induced a significant increase in the mRNA expression of tumor necrosis factor-alpha (TNF-α), interleukin-6 (IL-6) and interleukin-1β (IL-1β). However, a marginal or no induction of transcripts for these inflammatory cytokines was observed in macrophages derived from MyD88-deficient mice (14, 15). Interestingly, the TLR2 ligand PGN, TLR5 ligand fiagellin and TLR9 ligand CpG-DNA also failed to activate the cellular immune response and produce proinflammatory cytokines in MyD88-deficient mice (15–19), suggesting that MyD88 is critical for the signal transduction of TLR2, TLR4, TLR5 and TLR9. In addition to TLRs, IL-1R signaling, another critical mediator of innate immunity, also requires MyD88. The TIR domain of MyD88 interacts with the TIR domains of IL-1R1, and its death domain can directly bind the serine-threonine kinases IRAKs, providing the link between IL-1R1 and IRAKs to induce the activation of NF-κB and c-Jun N-terminal kinase (JNK) during pathogen challenge (12, 20). These observations revealed that MyD88-dependent signaling pathway plays a central role in the IL-1/Toll receptor-induced innate immune response.

Previous studies have demonstrated that MyD88 plays a central role in host defense against bacterial challenges (21, 22). The work by Takeuchi et al. found that MyD88-deficient mice were highly susceptible to *Staphylococcus aureus* infection and the production of immune cytokines TNF-α and IL-6 was not observed in peritoneal macrophages (23). In RAW macrophage cells, MyD88 is essential for the induction of a protective immune response to *Mycobacterium tuberculosis* infection through the regulation of TNF-α production in mice (22). In recent years, MyD88s have attracted considerable attention in aquatic animals due to their crucial roles in the innate immune systems. For instance, a fish MyD88 gene was identified from the Japanese eel and its expression level was significantly induced by *Edwardsiella tarda* infection in *Anguilla japonica* (24). *Ca*MyD88, the MyD88 homolog from Qihe crucian carp, was reported to play a critical role in the immune defense of *Carassius auratus* against *Aeromonas hydrophila* challenge (25). In miiuy croaker, it was found that MyD88-mediated NF-κB pathway participates in the regulation of the production of inflammatory cytokines in response to *Vibro harveyi* and LPS stimulation, and this pathway is negatively regulated by miR-214 (26). In shrimp, the MyD88-dependent signaling pathway was essential for the defense response of *Vibrio anguillarum* and *Micrococcus lysodeikticu* infection (27). Recently, Yipeng Ren et al. reported that MyD88 is involved in inducing the expression of immune-related genes, including C-type lysozyme and antimicrobial peptide, in *Cyclina sinensis* (28). In summary, these previous reports indicated that MyD88 plays a key role in the innate immune response to pathogenic challenge in aquatic animals (including fish, shrimp, shellfish, etc.).

*Anodonta woodiana* is one of the most economically important farmed freshwater shellfish. However, bacterial diseases seriously harm its health breeding and cause serious economic loss. Similar to other shellfish, *A. woodiana* belongs to invertebrates, which exclusively rely on the innate immune system to resist various external pathogenic factor invasions and maintain a normal standard of physical health. Investigating the molecular characteristics and immune function of the *A. woodiana* MyD88 gene, a key molecule in the innate immunity system (12), is important to understand the immune defense mechanism of freshwater mussels against pathogen infection. Therefore, a shellfish MyD88 homolog, *Aw*MyD88, was cloned and characterized from *A. woodiana*, and its mRNA profiles upon exposure to immune challenge were analyzed by quantitative real-time PCR (qRT-PCR). In addition, *Aw*MyD88 was overexpressed in human embryonic kidney 293T (HEK293T) cells to determine its intracellular localization and function in signal transduction. The findings of the present study will contribute to a better understanding of the function of the MyD88-dependent signaling pathway in freshwater shellfish.

## Materials and Methods

### Experimental Animals, Immune Challenge, and Sample Collection

The experimental *A. woodiana* (averaging 100 mm in shell height) were collected from a farm in Zhanjiang, China and maintained in 400-L laboratory aquarium tanks with continuously aerated freshwater at 24 ± 1°C for one week before processing. The healthy state of *A. woodiana* was confirmed by tissue morphology observation with HE staining. Bacteria strains were isolated from blood and digestive gland of shellfish and identified by their morphological and biochemical characteristics and 16S rDNA gene sequence. After confirmation of non-pathogen infection, seven tissues (hemocytes, muscle, gill, mantle, heart, foot and hepatopancreas) were collected from healthy *A. woodiana* for tissue expression profile analysis. The collected samples were quickly frozen in liquid nitrogen, and then stored at -80°C until RNA isolation.

For the *in vitro* immune challenge experiments, 150 mussels were randomly separated into three groups, including the control group, lipopolysaccharide (LPS) group and *A. hydrophila* group. Each group contained 50 individuals maintained in aerated tanks. Gram negative bacterium *A. hydrophila* were cultured in Luria-Bertani (LB) with shaking at 28°C. The shellfish in the control group received an injection with 100 μl of sterile phosphate buffer solution (PBS) (10 mM Na_2_HPO_4_, 140 mM NaCl, 2.7 mM KCl, 1.8 mM KH_2_PO_4_, pH 7.4). In the treatment groups, shellfish received an injection of 100 μl LPS (10 μg/ml in sterile PBS; Sigma Aldrich, USA) or *A. hydrophila* (1 × 10^7^ cfu/ml suspended in sterile PBS). After injection, *A. woodiana* were randomly sampled from each group at 0, 3, 6, 12, 24, 48 and 72 h post-stimulation, and the hemocytes were collected for RNA isolation. To further understand the possible immune function of *Aw*MyD88, an RNAi experiment was performed using the T7 RiboMAX™ Express RNAi System (Promega, USA) according to the manufacturer’s instructions. Healthy mussels were injected with 100 μg of ds*Aw*MyD88 (*Aw*MyD88-dsRNA, experimental group) or dsEGFP (EGFP-dsRNA, control group), equal volume of PBS (pH 7.4, blank group) into the adductor muscle. After injection, the mussels were returned to water tanks, and hemocytes were randomly collected from mussels of each group at one week postinjection. RNAi efficiency of *Aw*MyD88 in hemocytes was assessed by qRT-PCR. For the bacterial challenge experiments, 60 mussels were randomly divided into three treatment groups (PBS group, *A. hydrophila* group, *A. hydrophila* + ds*Aw*MyD88 group). After 12 h of treatment, the hemocytes from each group were obtained for analysis of the regulatory function of *Aw*MyD88 on the *A. hydrophila* induced immune response. All the samples were frozen immediately in liquid nitrogen and stored at -80°C.

All experiments were performed according to the recommendations of the Guidance of the Care and Use of Laboratory Animals in China. The research presented in this manuscript was approved by the on the Animal Ethics Committee of Changsha University.

### Hemocytes Culture and Immune Challenge

For the *in vitro* immune challenge experiments, primary cultured hemocytes of *A. woodiana* were prepared as described in previous reports (29, 30) and challenged as follows: the hemocytes were inoculated into 6-well cell culture plates (Corning, USA) at a density of 6 × 10^6^ cells/ml. The experimental cells were treated with *A. hydrophila* (1.0 × 10^7^ cfu/mL) and LPS (10 μg/mL, Sigma) to measure their effects on the expression of *Aw*MyD88 in *A. woodiana*. Control cultures were incubated with an equal volume of PBS. After 0, 3, 6, 12, and 24 h of treatment, hemocytes from three replicates were collected and washed with PBS for RNA extraction. The samples were stored in liquid nitrogen until further use.

### Total RNA Isolation and cDNA Synthesis

Total RNA was extracted from tissue distribution and immune experiment samples using the RNAiso plus (TaKaRa, Japan) reagent following the vendor’s protocol. The concentration and quality of isolated RNA were measured using a NanoDrop 2000 (Thermo Fisher, USA) and electrophoresis on 1.5% agarose gels, respectively. Then, the RNA samples were processed with gDNA Eraser (TaKaRa, Japan) to eliminate genomic DNA contamination. First-strand cDNA was synthesized with 1 μg total RNA using the PrimeScript™ 1st Strand cDNA Synthesis Kit (Takara, Japan) and PrimeScript™ RT Reagent Kit with gDNA Eraser (Takara, Japan) according to instructions. The obtained cDNA was used for subsequent gene cloning and expression analysis. The cDNA mix was diluted to 1:10 before further experiments.

### Gene Cloning

The cDNA sequence of *Aw*MyD88 was obtained by reverse transcription - polymerase chain reaction (RT-PCR). Gene specific primers (**Supplementary Table S1**) were designed using Primer Premier 5.0 software according to the sequences of the *Aw*MyD88 gene from our constructed *A. woodiana* cDNA library. The open reading frame (ORF) sequence of *Aw*MyD88 was amplified using LA Taq DNA polymerase (Takara) in a 25 μl reaction volume containing 15.25 µl of dH_2_O, 4 µl of dNTP Mixture (2.5 mM each), 2.5 µl of 10×LA Taq Buffer II (Mg^2+^ Plus), 1 µl of each primer (10 μM), 0.25 µl of LA Taq and 1 µl of cDNA template. The PCR amplification program was performed according to the manufacturer’s protocol. Briefly, 35 cycles of denaturation at 94°C for 30 s, annealing at 60°C for 30 s and extension at 72°C for 2 min were conducted for amplification. The PCR amplification products were separated using 1.0% agarose gel electrophoresis with Goldview nucleic acid stain, purified with a TaKaRa Agarose Gel DNA Purification Kit Ver.2.0 and subsequently cloned into the pMD19-T plasmid vector (Takara, Japan) according to the manufacturer’s instructions. Positive colonies were screened and further confirmed by DNA sequencing.

### Bioinformatic Analysis

Nucleotide and amino acid sequences were analyzed using the BLAST tool at the National Center for Biotechnology Information (NCBI) (http://blast.ncbi.nlm.nih.gov/Blast.cgi) and the Expert Protein Analysis System (http://www.expasy.org/). The open reading frames (ORFs) were predicted with online website (https://www.ncbi.nlm.nih.gov/orffinder/). Protein domain analysis was performed using the SMART program (http://smart.embl-heidelberg.de/). The identity and similarity between these amino acid sequences were calculated using MatGAT2.02 software (31). Nuclear localization signals (NLSs) and transmembrane domain were predicted by the cNLS Mapper (http://nls-mapper.iab.keio.ac.jp/cgi-bin/NLS_Mapper_form.cgi) and TMHMM-2.0 (https://services.healthtech.dtu.dk/service.php?TMHMM-2.0), respectively. The three-dimensional structures of *Aw*MyD88 were modeled using Swiss-Model software (https://swissmodel.expasy.org/) (32). Multiple sequence alignment was conducted using the CLUSTALW program (http://www.genome.jp/tools/clustalw/), and GeneDoc software was employed to visualize the results. The neighbor-joining (NJ) phylogenetic tree was constructed *via* 1000 bootstrap replications using MEGA 5.0 (33). The GenBank accession numbers corresponding to the MyD88 protein sequences examined are as follows: AAB49967.1 [*Homo sapiens*], AAC53013.1 [*Mus musculus*], NP_001014404.1 [*Bos taurus*], ABW74617.1 [*Sus scrofa*], XP_003992302.1 [*Felis catus*], XP_001488599.3 [*Equus caballus*], NP_001124153.1 [*Macaca mulatta*], PNI73973.1 [*Pan troglodytes*], NP_997979.2 [*Danio rerio*], ADE20131.1 [*Cyprinus carpio*], QXL90246.1 [*Ctenopharyngodon idella*], QBH74502.1 [*C. auratus*], NP_001130017.1 [*Salmo salar*], ADM25313.1 [*Siniperca chuatsi*], AEY83971.1 [*Lateolabrax japonicus*], ADZ44623.1 [*Oplegnathus fasciatus*], AYP28178.1 [*Tachysurus fulvidraco*], AFP49302.1 [*Penaeus vannamei*], AIS92906.1 [*P. monodon*], KAG7175226.1 [*Homarus americanus*], AFZ95001.1 [*Scylla serrata*], AGT21377.1 [*Eriocheir sinensis*], QNT17952.1 [*Macrobrachium rosenbergii*], RXG59101.1 [*Armadillidium vulgare*], AHB62785.1 [*Hyriopsis cumingii*], AEF32114.1 [*Ruditapes philippinarum*], AHK60398.1 [*Haliotis diversicolor*], AKN04686.1 [*Mizuhopecten yessoensis*], AFX68459.1 [*Crassostrea gigas*], ABB76627.1 [*Azumapecten farreri*], CAC5373926.1 [*Mytilus coruscus*], AIZ97751.1 [*C. sinensis*], ON082068 [*Anodonta woodiana*].

### Quantitative Real-Time PCR (qRT-PCR) Analysis

The mRNA expression levels of target genes were investigated by qRT-PCR on a Bio-Rad CFX96™ Real-time PCR Detection System (Bio-Rad, USA). Briefly, *Aw*β-actin was amplified with the specific primers, *Aw*β-actin-F and *Aw*β-actin-R (**Supplementary Table S1**), and this gene serve as an endogenous control. The qRT-PCR reactions were performed in a 16 μl mixture containing 5.68 μl of RNase-free water, 1 μl of diluted cDNA template, 8 μl of 2×SYBR Premix Ex Taq II (Tli RNaseH Plus) (Takara, Japan), 0.5 μl of each primer and 0.32 μl 50×ROX Reference Dye II. The following qRT-PCR program was employed: 95°C for 5 min, followed by 40 cycles at 95°C for 10 s and 60°C for 45 s. To confirm the specificity of PCR products, the dissociation curve analysis of amplification products was performed at the end of each PCR. Relative mRNA expression levels of target genes were analyzed by comparative Ct method (2^-ΔΔCT^) and exported into a Microsoft Excel spreadsheet for subsequent data analysis.

### Construction of Expression Vectors

Eukaryotic expression vectors, including pCMV-N-Flag-*Aw*MyD88 (*Aw*MyD88-Flag), pCMV-N-Flag-*Aw*TLR (*Aw*TLR-Flag) and pEGFP-N1-*Aw*MyD88 (*Aw*MyD88-GFP), were constructed for mammalian cell transfections using the ClonExpress^®^ II One Step Cloning kit (Vazyme, China) according to the manufacturer’s protocol. In brief, the primer pairs *Aw*MyD88-F3/R3, *Aw*TLR-F2/R2 and *Aw*MyD88-F4/R4 (**Supplementary Table S1**) were designed using Primer Premier 5.0 software based on the cDNA sequences of *Aw*MyD88 and *Aw*TLR. The ORFs of *Aw*MyD88 and *Aw*TLR were amplified, and then ligated into eukaryotic expression vectors. The recombinant plasmids were identified and verified by colony PCR and DNA sequencing. Upon sequence verification, the positive recombinant plasmids were transformed into competent *E. coli DH5α* cells and were isolated from overnight bacterial cultures on 50 ml of LB liquid medium supplemented with kanamycin (100 μg/mL) at 37°C using EndoFree Plasmid Kits (Qiagen, Germany) according to the manufacturer’s instructions.

### Cell Culture and Transient Transfection

Due to the lack of available established cell lines for freshwater shellfish, human embryonic kidney 293T (HEK293T) cells were used for subcellular localization and luciferase reporter analyses. The cells were cultured at 37°C in a humidified incubator with 5% CO_2_ in DMEM (Gibco, USA) containing 10% (v/v) fetal bovine serum (Gibco, USA), 10^5^ U/L penicillin and 100 mg/L streptomycin (Gibco, USA).

For transfection, cells were seeded in culture plates and cultured for 12 h to attain 70-80% confluence at the time of transfection. Before transfection, the cells were washed with PBS, and the medium was replaced with Opti-MEM (Invitrogen, USA). The prepared recombinant vectors were transiently transfected into HEK293T cells using Lipofectamine 2000 Transfection Reagent (Invitrogen, USA) following the manufacturer’s instructions. After 4-6 h, the medium was replaced with a complete medium containing 10% FBS.

### Subcellular Localization

For subcellular localization analysis of *Aw*MyD88, cells were seeded on sterile coverslips at 1 × 10^5^ cells/well in 6-well plates overnight growth prior to transfection. The endoFree plasmid pEGFP-N1-*Aw*MyD88 (1 µg/well) or pEGFP-N1 (1 µg/well) was transfected into HEK293T cells with Lipofectamine 2000 in serum-free culture medium. Forty-eight hours after transfection, the transfected cells were washed with PBS and then fixed with 4% (v/v) paraformaldehyde (PFA). After washing with PBS, the cells were treated with PBST and stained with 4,6-diamino-2-phenylindole (DAPI). The coverslips were then washed and transferred to glass slides containing Antifade Mounting Medium (Beyotime). Finally, the samples were visualized using fluorescence microscopy (Leica, Germany).

### Luciferase Reporter Gene Assay

For dual-luciferase reporter assays, the recombinant plasmid *Aw*MyD88-Flag/*Aw*TLR-Flag (0, 300, 600 ng/well) was cotransfected with pNF-κB-Luc/pAP-1-Luc (100 ng/well) and the pRL-TK Renilla luciferase plasmid (20 ng/well) into HEK293T cells plated in 96-well plates. At 48 h posttransfection, the HEK293 T cells in 96-well plates were washed twice with PBS and lysed. Firefly and Renilla luciferase activities were measured using a luciferase reporter assay system (Promega, USA) according to the manufacturer’s instructions. The relative luciferase activity of each trial was calculated as the ratio of firefly luciferase activity to Renilla luciferase activity. The pRL-TK plasmid was used as the internal control and the pCMV-N-Flag plasmid served as the negative control. Each sample was analyzed in triplicate.

### Statistical Analysis

The results from qRT-PCR and Luciferase reporter gene assay were shown as the mean ± standard deviation (SD) of triplicate data. The data were analyzed for significant differences using analysis of variance (ANOVA) with LSD or Duncan *post hoc* test at the level of *P* < 0.05 or *P* < 0.01 using SPSS Statistics 17.0 (SPSS Inc., Chicago, USA).

## Results

### cDNA Cloning and Sequence Analysis of *Aw*MyD88

**Figure 1:**
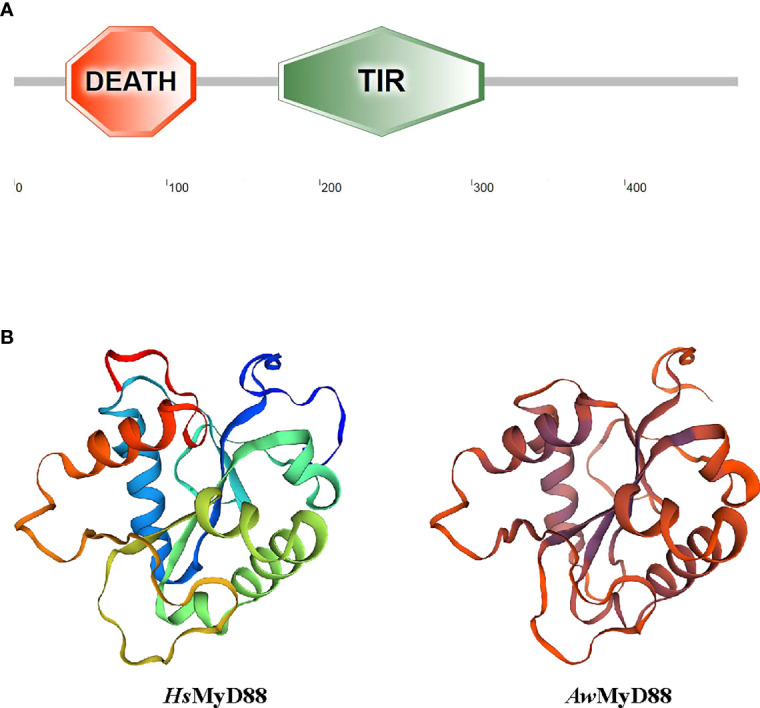
Domain organization and three-dimensional structure of *Aw*MyD88. **(A)** Domain analysis of *Aw*MyD88 was performed using the SMART tool. **(B)** Tertiary structures of *Hs*MyD88 and *Aw*MyD88 were predicted by the SWISS-MODEL program.

Using RT-PCR amplification, the cDNA sequence of *Aw*MyD88 was cloned from *A. woodiana* and submitted to GenBank with accession number ON082068 (**Figure S1**). The open reading frame of *Aw*MyD88 cDNA was 1,425 bp in length which encoded a 474 amino acid polypeptide with a predicted molecular weight of 53.93 kDa and pI of 4.93. SMART analysis showed that the *Aw*MyD88 protein contained a conserved DEATH domain and a TIR domain (**Figure 1A**). No nuclear localization signal (NLS) sequences and transmembrane domains were observed in the *Aw*MyD88 protein, indicating that *Aw*MyD88 is a soluble cytoplasmic protein. The three-dimensional (3D) molecular modeling of *Aw*MyD88 was predicted using the solution NMR structure of human MyD88 (2js7.1.A) as the template (**Figure 1B**). The results indicated that *Aw*MyD88 shared a similar 3-D structure with MyD88 from *Homo sapiens*.

### Homology Alignment and Phylogenetic Analysis

**Figure 2:**
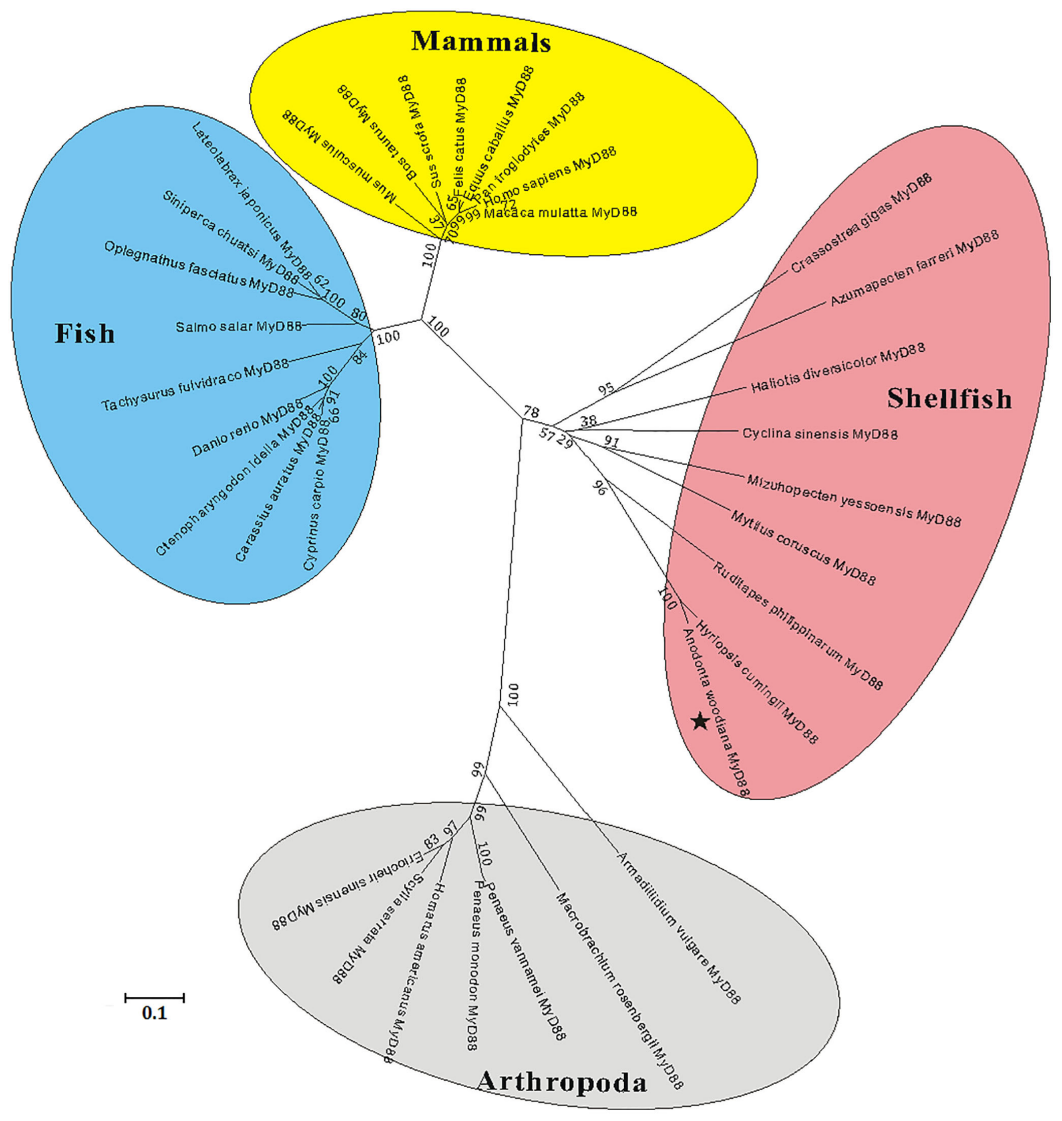
Phylogenetic analysis of MyD88s by MEGA 5.0 software based on the neighbor-joining method. The numbers at the nodes represent bootstrap values for 1000 replications and the bar (0.1) indicates genetic distance. *Aw*MyD88 is labeled with a black pentagram.

**Figure 3:**
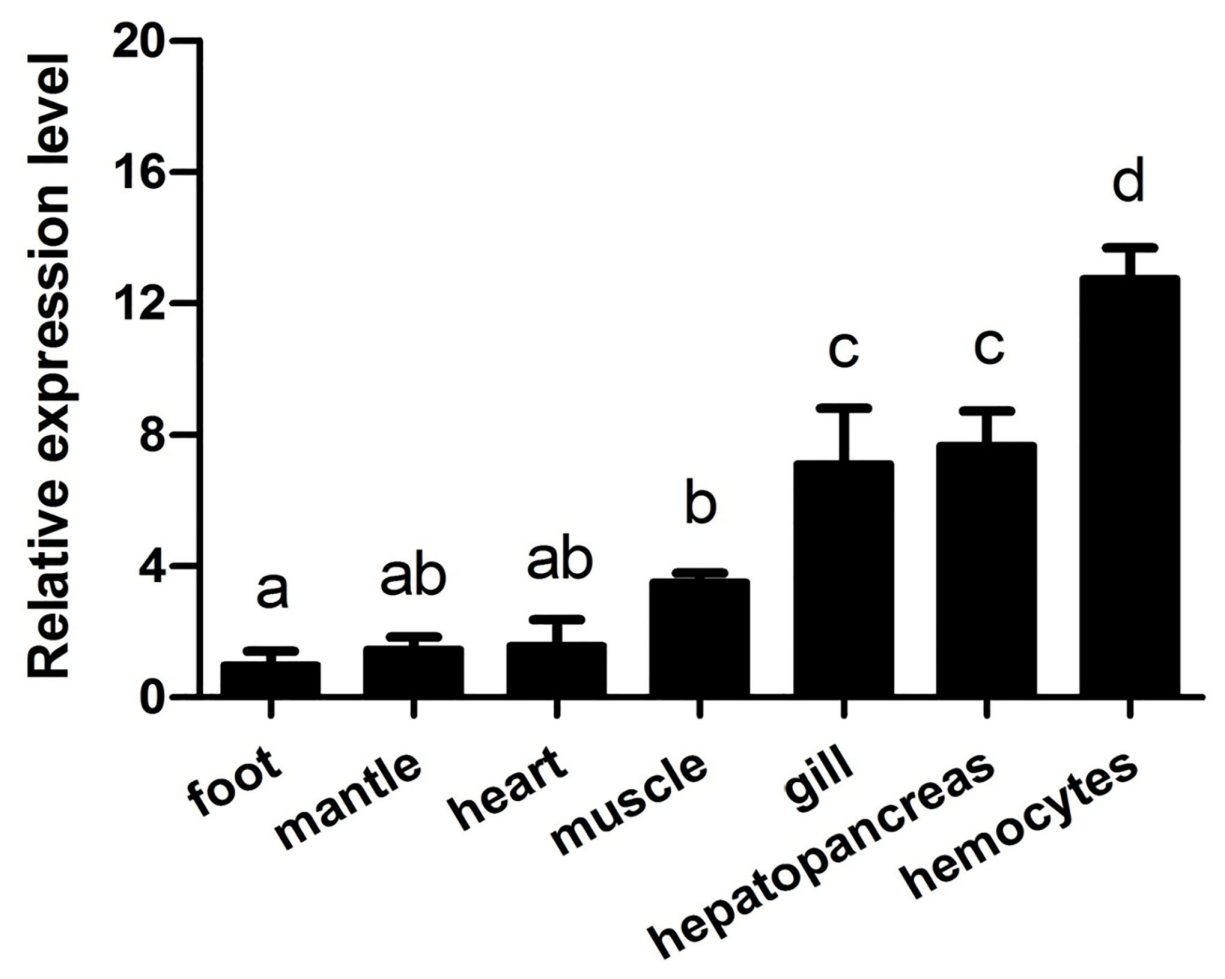
Expression profiles of *Aw*MyD88 in different tissues of *A. woodiana* were determined by qRT-PCR. β-actin expression served as an internal control. The relative expression of *Aw*MyD88 is presented as the fold change for the foot. Data are shown as mean ± S.E. (*N* = 3). Different letters above the bars indicate significant differences (*P* < 0.05).

A multiple sequence alignment analysis based on the amino acid sequences of MyD88s showed that the functional domains of *Aw*MyD88, including a typical Death domain, a TIR domain and three boxes, displayed relative conservation with those of MyD88 orthologs from other species (**Figure S2A**). Further pairwise sequence results using MatGAT2.02 software found that *Aw*MyD88 shared different degrees of sequence similarity (S) and identity (I) with other known MyD88s, including 37.1% (S) and 24.1% (I) with *H. sapiens*, 37.1% (S) and 23.0% (I) with *M. musculus*, 38.2% (S) and % 24.4(I) with *B. taurus*, 38.4% (S) and 24.5% (I) with *D. rerio*, 38.2% (S) and 25.3% (I) with *C. carpio*, 38.2% (S) and 24.6% (I) with *C. idella*, 37.1% (S) and 24.7% (I) with *C. auratus*, 46.9% (S) and 25.3% (I) with *P. vannamei*, 91.2% (S) and 84.5% (I) with *H. cumingii*, 61.2% (S) and 39.9% (I) with *R. philippinarum*, 58.2% (S) and 37.0% (I) with *M. yessoensis* (**Figure S2B**). Among them, *Aw*MyD88 displayed the highest identity and similarity with the MyD88 ortholog from *H. cumingii*. A phylogenetic tree was constructed using the NJ method based on the amino acid sequences of *Aw*MyD88 and thirty-five other species (**Figure 3B**). The results showed that these MyD88 homologs could be divided into four groups, consisting of mammals, arthropod, fish and shellfish branches, in which *Aw*MyD88 is located in shellfish branches and clustered together with *H. cumingii* MyD88 (**Figure 2**).

### Tissues Distribution and Expression Profiles of *Aw*MyD88 in Response to *A. hydrophila* and LPS Challenge

The expression levels of *Aw*MyD88 in various tissues (gill, mantle, heart, muscle, hemocytes, foot and hepatopancreas) were examined by qRT-PCR. As shown in **Figure 3**, *Aw*MyD88 was ubiquitously expressed in all the tested tissues, with the highest expression levels noted in hemocytes, moderate expression levels in gill and hepatopancreas, and relatively low levels in muscle, heart, mantle and foot.

**Figure 4:**
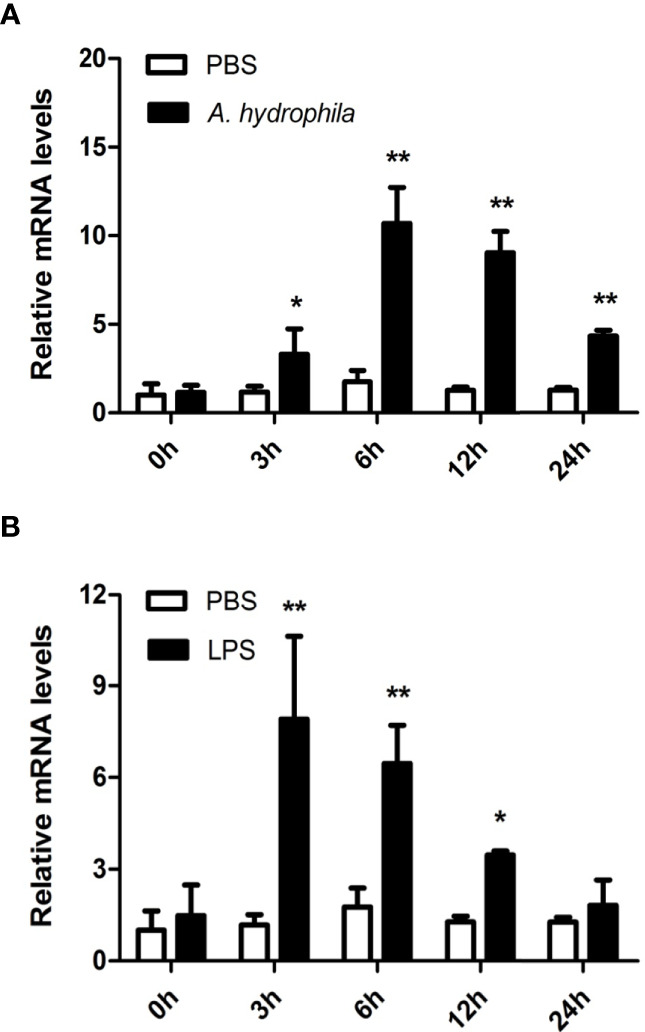
Expression profiles of *Aw*MyD88 in primary cultured hemocytes infected with *A. hydrophila* **(A)** or LPS **(B)** *in vitro*. The β-actin gene was used as the internal control to calibrate the cDNA template for all the samples. The expression level of the control group (PBS) at 0 h postchallenge was set as 1.0, and the results are shown as mean ± S.E. (*N* = 3). Significant differences between the immune challenge and control groups are indicated by the asterisks (* and ** represent *P* < 0.05 and *P* < 0.01, respectively).

Hemocytes are considered the main immunological cells in bivalve mollusks and rapidly respond to pathogenic invasion. In this study, the expression patterns of *Aw*MyD88 in primary cultured hemocytes response to bacterial infection *in vitro* were investigate. As shown in **Figure 4**, the expression levels of *Aw*MyD88 in hemocytes were obviously increased in a time-dependent manner after exposure to *A. hydrophila* and LPS. When challenged with *A. hydrophila*, *Aw*MyD88 mRNA expression initially significantly increased at 3 h postchallenge (2.8-fold; *P* < 0.05), reaching the peak value at 6 h postchallenge (6.1-fold; *P* < 0.01). Similar to the *A. hydrophila* challenge experiment, hemocytes treatment with LPS also first significantly upregulated the transcripts of *Aw*MyD88 at 3 h post-stimulation (6.7- fold; *P* < 0.01), and then gradually declined from 6 h to 24 h post-stimulation.

**Figure 5:**
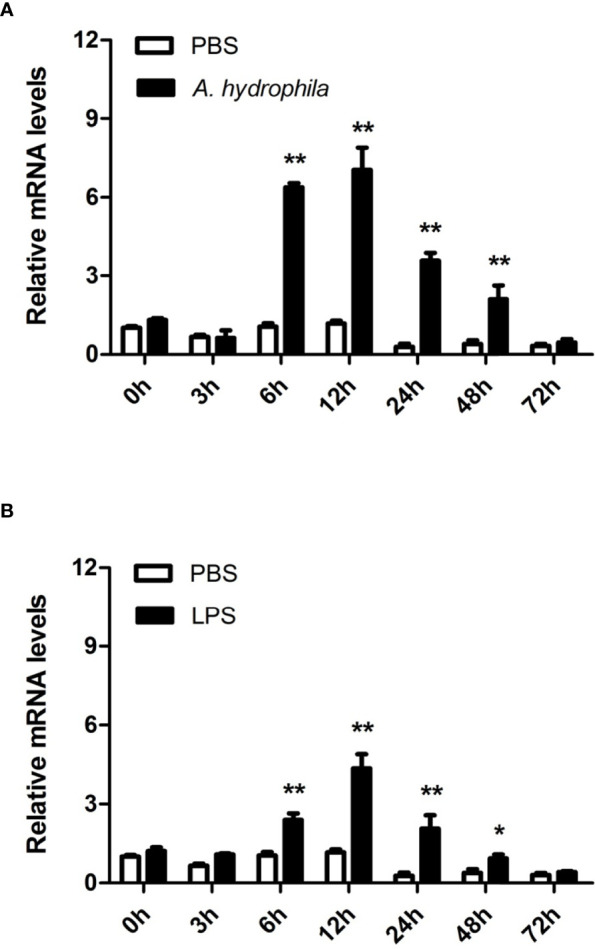
Expression profiles of *Aw*MyD88 in hemocytes infected with *A. hydrophila* **(A)** or LPS **(B)** *in vivo*. The β-actin gene was used as an internal reference gene to normalize the expression level. The expression level of the control group (PBS) at 0 h postchallenge was set as 1.0, and the results are presented as mean ± S.E. (*N* = 3). Significant differences between the immune challenge and control groups are indicated by the asterisks (* and ** represent *P* < 0.05 and *P* < 0.01, respectively).

To further unravel the immune function of *Aw*MyD88 in hemocytes, the expression profile of *Aw*MyD88 was also examined in hemocytes of *A. woodiana* at different time points after challenge with *A. hydrophila* and LPS. Following injection with *A. hydrophila* and LPS, a similar variation in the time-dependent expression profile of *Aw*MyD88 mRNA was observed in hemocytes of *A. woodiana* (**Figure 5**). In brief, the transcript levels of *Aw*MyD88 showed no significant changes until 6 h postinjection (*P* < 0.05), peaked at 12 h postinjection (*P* < 0.01), and then returned to normal levels at 72 h postinjection (*P* > 0.05). These results indicated that *Aw*MyD88 may be involved in the immune defense against bacterial infections in hemocytes of *A. woodiana*.

### Expression Profiles of MyD88 Signaling Pathway Genes in Response to *A. hydrophila* Challenge

**Figure 6:**
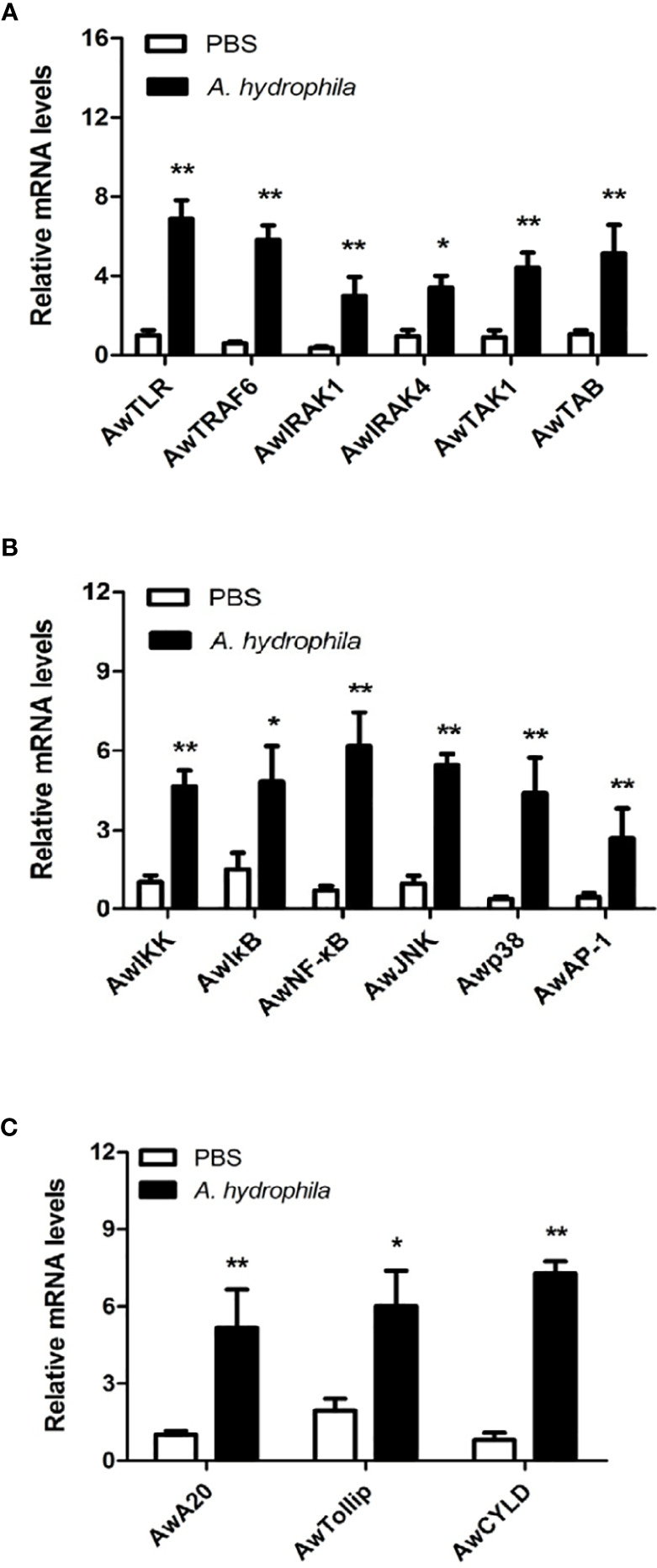
Expression profiles of *Aw*MyD88 pathway signaling molecules **(A, B)** and negative regulatory proteins **(C)** in response to *A. hydrophila* challenge. The β-actin gene served as an internal reference gene to normalize the expression level. Each bar represents the mean of the normalized expression levels of the replicates (*N* = 3). Significant differences between the immune challenge and control groups are indicated by the asterisks (* and ** represent *P* < 0.05 and *P* < 0.01, respectively).

To investigate the possible role of the MyD88-dependent signaling pathway in innate immunity, the mRNA expression levels of numerous immune related genes, including signaling molecules (*Aw*TLR, *Aw*TRAF6, *Aw*IRAK1, *Aw*IRAK4, *Aw*TAK1, *Aw*TAB, *Aw*IKK, *Aw*IκB, *Aw*JNK and *Aw*p38), transcription factors (*Aw*NF-κB and *Aw*AP-1) and negative regulatory molecules (*Aw*A20, *Aw*Tollip and *Aw*CYLD) were determined after *A. hydrophila* challenge in hemocytes of *A. woodiana.* The qRT-PCR results showed that the mRNA expression levels of all selected genes in the hemocytes were significantly increased by *A. hydrophila* infection at 12 postinjection (*P* < 0.05), suggesting that the MyD88***-*** dependent signaling pathway could be activated by *A. hydrophila* challenge and may play an important role in the immune defense response to bacterial infections (**Figure 6**).

### Effects of *Aw*MyD88 Knockdown on the Expression of Immune Related Genes

**Figure 7:**
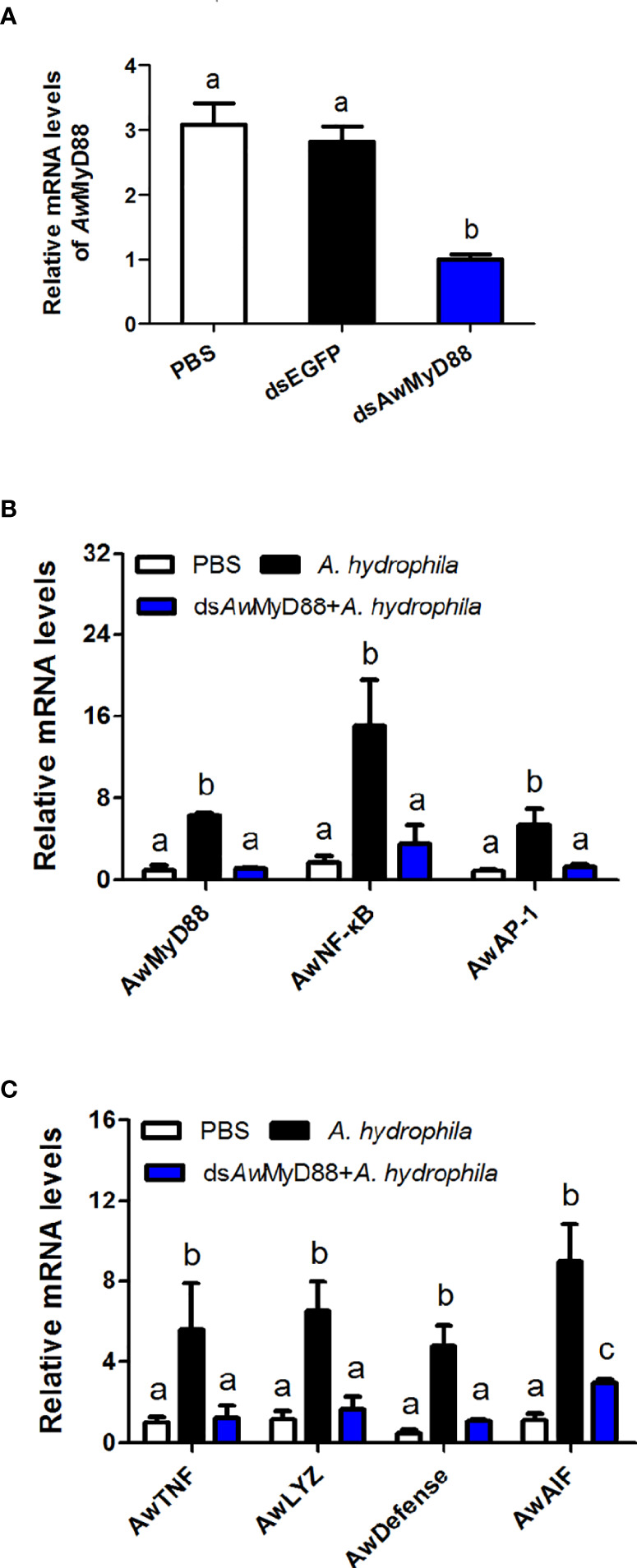
Effects of *Aw*MyD88 knockdown on the expression of *Aw*MyD88 **(A)** and immune-related genes **(B, C)** in hemocytes of *A. woodiana*. The β-actin gene served as an internal reference gene to normalize the expression level. Each bar represents the mean of the normalized expression levels of the replicates (*N* = 3). Significant differences between the immune challenge and control groups are indicated by different letters (P < 0.05).

To determine the regulatory role of *Aw*MyD88 on the innate immunity system of *A. woodiana*, the expression patterns of immune related transcription factors (*Aw*NF-κB and *Aw*AP-1) and effectors (*Aw*TNF, *Aw*LYZ, *Aw*Defense and *Aw*AIF) were detected by qRT-PCR after *Aw*MyD88 knockdown. As shown in **Figure 7A**, transcript levels of *Aw*MyD88 were significantly decreased in the ds*Aw*MyD88 injection group compared to that in the control group (dsEGFP) and the blank group (PBS), suggesting a relatively high knockdown efficiency of *Aw*MyD88 in hemocytes of *A. woodiana*. In *A. hydrophila* challenge experiments, it was found that the knockdown of *Aw*MyD88 could downregulated the mRNA expression of the downstream transcription factors NF-κB and AP-1 in hemocytes of *A. woodiana* (**Figure 7B**). Additionally, the transcript levels of immune defense genes, including TNF, LYZ, Defense and AIF, were significantly inhibited by *Aw*MyD88 RNAi in *A. woodiana* (**Figure 7C**). These findings further indicated that *Aw*MyD88 was essential for the innate immune response to bacterial infection in freshwater shellfish.

### Subcellular Localization of *Aw*MyD88

**Figure 8:**
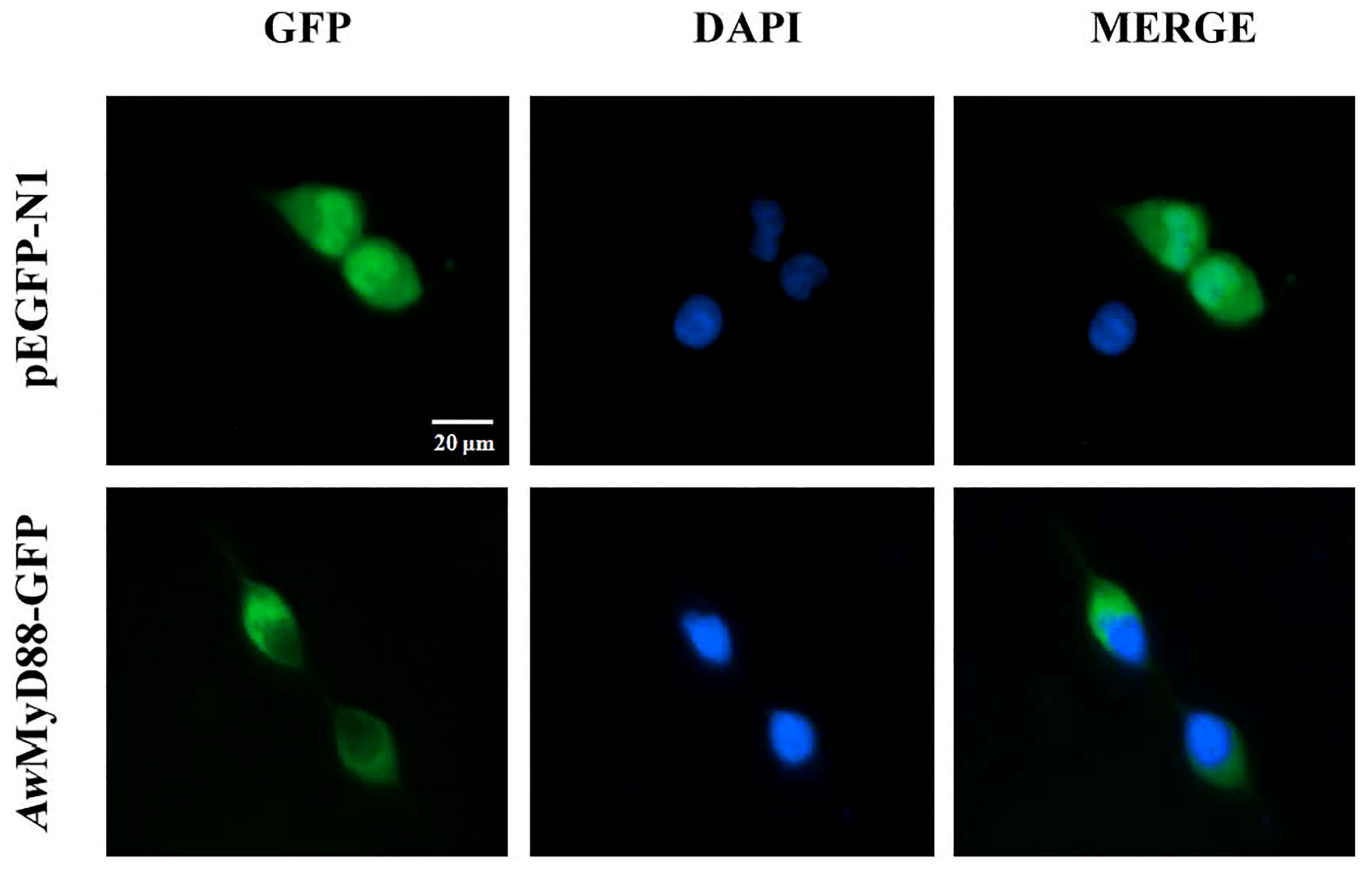
Subcellular localization of *Aw*MyD88-GFP in HEK293T cells. HEK293T cells were transiently transfected with *Aw*MyD88-GFP or pEGFP-N1 (control). At 48 h posttransfection, the nucleus was stained with DAPI, and the cells were imaged under a fluorescence microscope.

To investigate the subcellular localization of *Aw*MyD88, the recombinant plasmid *Aw*MyD88-GFP was constructed and transfected into HEK293T cells. The results showed that the cells harboring the control plasmid pEGFP-N1 showed green fluorescence both in the cytoplasm and nucleus (**Figure 8**). However, the *Aw*MyD88-GFP fusion protein was mainly distributed in the cytoplasm, implying a predominant cytoplasm localization of *Aw*MyD88 in HEK293T cells.

### Effects of *Aw*MyD88 Overexpression on the NF-κB and AP-1 Signaling Pathways

**Figure 9:**
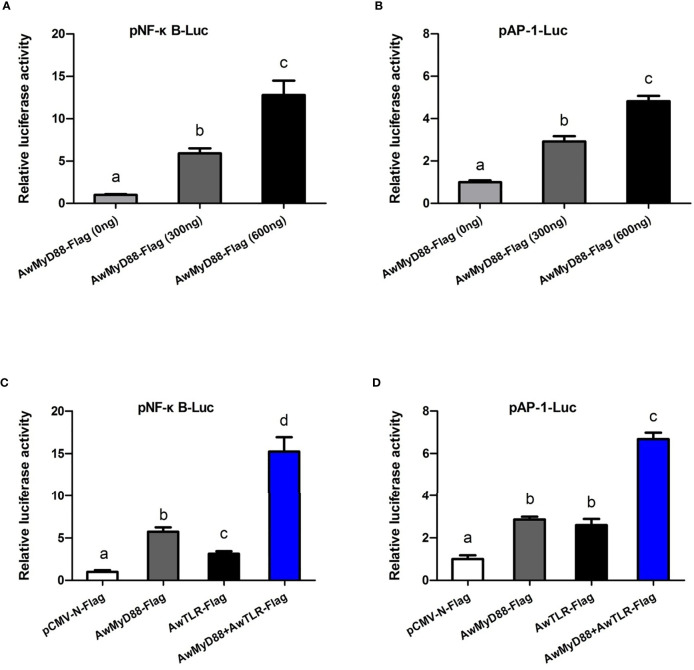
Effects of *Aw*MyD88 overexpression on the activity of the NF-κB **(A, C)** or AP-1 **(B, D)** reporter genes in HEK293T cells. The expression plasmids *Aw*MyD88-Flag/*Aw*TLR-Flag were cotransfected with NF-κB/AP-1 luciferase reporter plasmids and the pRL-TK Renilla luciferase plasmid into HEK293T cells using Lipofectamine 2000 Transfection Reagent. Firefly and Renilla luciferase activities were detected in cell lysates 48 h after transfection. Relative luciferase activities were calculated by normalizing to the pRL-TK value. The results are represented as the mean values of three independent experiments (*N* = 3), and error bars represent standard deviations. Different letters indicate significant differences compared with other groups (*P* < 0.05).

To explore whether *Aw*MyD88 can modulate the activation of the NF-κB and AP-1 signaling pathways, HEK293T cells were transfected with the plasmid *Aw*MyD88-Flag or empty control plasmid pCMV-N-Flag. As shown in **Figure 9A**, transfected *Aw*MyD88-Flag could significantly enhance NF-κB activation in HEK293T cells in a dose dependent manner, with a maximum increase of 12.8-fold relative to transfection of pCMV-N-Flag alone in HEK293T cells (*P* < 0.05). Similarly, *Aw*MyD88 overexpression significantly induced the activation of an AP-1-driven reporter gene in HEK293T cells (*P* < 0.05), which was also dependent on the dose of transfected *Aw*MyD88-Flag vector (**Figure 9B**). To further determine the possible mechanisms of *Aw*MyD88-induced NF-κB and AP-1 activation, *Aw*MyD88-Flag was cotransfected with *Aw*TLR-Flag into HEK293T cells for the dual-luciferase reporter assay. The results showed that the activation effects of cells transfected with *Aw*MyD88-Flag + *Aw*TLR-Flag on NF-κB-Luc or AP-1-Luc were significantly greater than those of cells transfected with *Aw*MyD88-Flag or *Aw*TLR-Flag alone (*P* < 0.05) (**Figures 9C, D**), suggesting that *Aw*MyD88-mediated activation of the NF-κB or AP-1 signaling pathway was positively regulated by upstream Toll-like receptors.

## Discussion

As a pivotal component of the innate immune system, MyD88 potentially participates in regulating the canonical TLR dependent activation of nuclear factor-κB and mitogen-activated protein kinases, which plays an important role in the host defense response against microbes (34). To date, MyD88s have been reported in a variety of aquatic animals (24, 25, 27); however, there is no experimental evidence regarding the presence of MyD88 in *A. woodiana*, one of the most important farmed freshwater shellfish in China. In this regard, we cloned and characterized the cDNA sequence of MyD88 gene from *A.woodiana* and explored its potential roles in innate immunity. Sequence analysis showed that *Aw*MyD88 contained a typical DD domain and a TIR domain, which is similar to other reported MyD88 proteins (21). Previous studies have demonstrated that the C-terminal TIR domain of MyD88 is responsible for its activation through TIR-TIR domain interactions with upstream TLRs (34, 35). It was shown that MyD88’s N-terminal death domain is essential for the recruitment and activation of downstream IRAK kinases to trigger immune response during pathogen challenge (35, 36). These conserved functional domains were observed in MyD88s from fish, shrimp and shellfish, implying that MyD88s may have a similar functional activation role in Toll-like receptor transduction. Homology alignment analysis showed that *Aw*MyD88 shares a relatively higher level of sequence similarity and identity with shellfish MyD88s than other reported MyD88 proteins. This finding was consistent with the results of the phylogenetic analysis, in which *Aw*MyD88 was found to be part of the shellfish branch and showed the closest evolutionary relationship with the *H. cumingii* homolog. Together, sequence alignment and phylogenetic analyses suggest that *Aw*MyD88 is a novel member of the shellfish MyD88 family.

Earlier studies showed that MyD88s are broadly expressed in a variety of tissues and organs in vertebrates (37, 38). For example, a ubiquitously expressed pattern of MyD88s was observed in many fish, including Northeast Chinese lamprey (39), spotted knifejaw (40), black carp (41), Japanese eel (24) and Qihe crucian carp (25). In invertebrates, MyD88 transcripts are expressed in various tissues of shrimp (27), crabs (42), echinoderms (43) and shellfish (21). In the present study, our qRT-PCR results showed that the mRNA expression of *Aw*MyD88 could be detected in all the tested tissues of *A. woodiana*, which was consistent with the tissue expression pattern of MyD88s in other shellfish (44, 45), indicating its broader, more generalized role in numerous physiological processes. Previous studies found that MyD88s were abundantly expressed in multiple immune-related tissues in aquatic animals. In fish, relatively high expression of MyD88 has been detected in the spleen of *Pelteobagrus fulvidraco* (46) and *C. auratus* (25), and intestine of *Pseudosciaena crocea* (38). In mollusks, MyD88s were also shown to be highly expressed in hemocytes, gill, mantle and hepatopancreas, which are important tissues for triggering the innate immune response against pathogens (44). In our study, the highest expression levels of *Aw*MyD88 were detected in hemocytes, which was consistent with the results in *Portunus trituberculatus* (42) and *C. sinensi* (28). It is generally believed that hemocytes are one of the key immune tissues in bivalve mollusks and play crucial roles in the recognition and elimination of bacterial pathogens *via* phagocytic activity and the production of lectins, antimicrobial peptides and lysosomal enzymes (47–50). Additionally, a relatively high transcript level of *Aw*MyD88s was observed in immune-related gill and hepatopancreas tissues, which are responsible for directly contacting the pathogens from the external environment and acting on the last immune barrier in the body (44, 51), respectively. Based on these findings, it can be hypothesized that *Aw*MyD88 may play a regulatory role in the innate immunity of *A. woodiana*.

Previous reports revealed that MyD88 plays a central role the in innate immune response to pathogenic challenge in vertebrates and invertebrates (38, 42). In mice, studies have shown that MyD88 participated in regulating the production of inflammatory cytokines in response to LPS, PGN, flagellin and CpG-DNA challenges (15–19). Underhill et al. found that MyD88 plays important roles in the induction of the host defense response during bacterial infection in RAW macrophage (22). Over the past decades, MyD88 genes were shown to be involved in the immune defense response against pathogen invasion in many aquatic animals (24, 27). As a freshwater shellfish cultured in rivers and lakes, *A. woodiana* is often exposed to challenging environments composed of various bacterial pathogens. It was necessary to investigate function of the MyD88 in the host defense system of *A. woodiana* in response to immune stimulation. In the present study, the temporal expression profile of *Aw*MyD88 in hemocytes was examined in response to bacterial LPS and *A. hydrophila* infections *in vitro* and *in vivo*. The results showed that *Aw*MyD88 had a stronger and broader response to bacterial challenges, and its transcript levels were significantly upregulated by these immune stimuli, implying that *Aw*MyD88 may be involved in host defense against bacterial infections. Additionally, it was found that *A. hydrophila* infection could significantly induce the mRNA expression levels of *Aw*MyD88-mediated signaling pathway-related genes in hemocytes, further indicating the involvement of *Aw*MyD88 in the host immune response against bacterial pathogens. To better understand the immune function of *Aw*MyD88, knockdown of *Aw*MyD88 was performed in hemocytes of *A. woodiana* by dsRNA-mediated RNAi. The results showed that *Aw*MyD88 knockdown could decrease the *A. hydrophila*-induced mRNA expression levels of immune-related transcription factors (NF-κB and AP-1) and effectors (TNF, LYZ, Defense and AIF) in hemocytes of *A. woodiana.* In recent years, knockdown experiments of MyD88s have been conducted in other aquatic animals (28, 41, 42). For example, Yipeng Ren et al. reported that the mRNA expression levels of *Cs*AMP and *Cs*C-LYZ were inhibited significantly in the *Cs*MyD88 dsRNA injection group compared to the control groups of *C. sinensi* (28). After the *Pt*-MyD88 gene was silenced in primary culture hemocytes, the expression of AMPs, including ALF1, hyastatin3, crustin1 and crustin3, was significantly suppressed in swimming crab *P. trituberculatus* (42). Together, these findings strongly supported that the MyD88-dependent signaling pathway was essential for host immune defenses against pathogenic invasion.

Toll-like receptor (TLR) signaling pathway is a key component of the immune system, which is responsible for recognizing invading pathogens and initiating an innate immune response (5, 6). In brief, TLRs could be activated by PAMPs stimulation and then recruit the intracellular adaptor protein MyD88 *via* a homophilic TIR-TIR interaction. Upon MyD88 activation, a series of signaling molecules, including TRAF6, IRAK1, IRAK4, TAK1, TAB, IKK, IκB, JNK and p38, were subsequently activated and finally induced the activation of the transcription factors NF-κB and AP-1 to regulate the expression of various immune genes involved in the defense response to bacterial challenge (6–8). Based on previous reports and our qRT-PCR results, a predicted diagram of the TLR-mediated MyD88-dependent signaling pathway in *A. woodiana* is presented in **Figure S3**. However, more experimental evidence is needed to support the predicted diagram of the MyD88-dependent signaling pathway in *A. woodiana*. Previously, MyD88 was shown to be mainly as a crucial cytosolic adapter protein involved in TLR signal transduction, which is evolutionarily conserved from invertebrates to vertebrates (11, 40, 52). Our fluorescence microscopy assay revealed that *Aw*MyD88-GFP was mainly distributed in the cytoplasm of HEK293T cells, suggesting that *Aw*MyD88 is a cytoplasmically localized protein that is consistent with its function as an adaptor protein for signal transduction in the TLR pathway. Similar results were reported in spotted knifejaw (*O. punctatus*), where OppMyD88 distribution was observed in the cytoplasm of HEK293T cells (40). Recently, the cellular localization of MyD88 in Japanese eel was detected in the cytoplasm of EPC cells (24). Interestingly, a MyD88 homolog from Northeast Chinese lamprey (*L. morii*) was localized in both the nucleus and cytoplasm of HEK293T cells (39). However, the exact molecular mechanism remains unclear and requires further investigation.

In mammals, the transcription factor NF-κB can be activated by the upstream TLR signaling pathway and serve as a pivotal regulator of immune, inflammatory and acute phase responses during pathogenic challenges (53, 54). As a conserved signaling intermediate in Toll pathways, MyD88s were shown to be involved in TLR-induced NF-κB activation (55–57). In boney fish, overexpression of MyD88s from *O. punctatus* (40) and *Epinephelus coioides* (58) could activate the NF-κB signaling cascade in HEK293T cells. The function of MyD88s in NF-κB activation has also been reported in aquatic invertebrates, such as *Holothuria leucospilota* (59), *H. discus discus* (60), *Pinctada fucata martensii* (45) and *M. coruscus* (44). In addition to activating NF-κB, MyD88 also modulates the activation of the AP-1 signaling pathway, which is essential for regulating the production of immune effectors (57, 61). These previous findings suggest that the MyD88-mediated NF-κB and AP-1 pathways were conserved from invertebrates to vertebrates. To determine the possible role of *Aw*MyD88 on the NF-κB/AP-1 signaling cascades, expression plasmids of *Aw*MyD88 were cotransfected with the luciferase reporter gene into HEK293T cells. The dual luciferase reporter assays showed that the overexpression of *Aw*MyD88 could significantly induce the activation of the NF-κB and AP-1 luciferase reporters in a dose-dependent manner, suggesting that *Aw*MyD88 acts as a positive regulator of the NF-κB and AP-1 signalling pathway. Similar overexpression experiments were performed in tropical sea cucumber where *HL*MyD88 significantly enhanced the activity of NF-κB and AP-1 luciferase reporters compared to the control in HEK293T cells (59). Additionally, our dual luciferase reporter assays found that *Aw*MyD88 could significantly enhance the TLR-induced activity of the NF-κB and AP-1 reporter genes, implying that *Aw*MyD88 may associate with *Aw*TLR in the activation of the NF-κB and AP-1 in HEK293T cells. Combined with the previous data of *Aw*MyD88 expression in response to bacterial challenges, it is hypothesized that the MyD88-mediated NF-κB/AP-1 signaling pathway is essential for innate immunity of *A. woodiana*.

In conclusion, the present study is the first to report the presence of a functional *Aw*MyD88 signaling pathway in the freshwater calm *A. woodiana*. Bioinformatic analyses indicated that *Aw*MyD88 contained conserved characteristic features of homologous proteins and shared evolutionary relatedness with its freshwater mollusk counterparts. The qRT-PCR results revealed that *Aw*MyD88 was widely expressed in various tissues and that its expression levels could be significantly induced by *A. hydrophila* and LPS challenge in hemocytes of *A. woodiana.* Gene knockdown experiments showed that *Aw*MyD88 was involved in regulating the expression of immune related genes during *A. hydrophila* infection. Overexpression analysis found that *Aw*MyD88 was mainly distributed in the cytoplasm and could effectively trigger activation of the NF-κB and AP-1 signaling pathways in HEK293T cells. These findings suggest that the *Aw*MyD88 dependent signaling pathway is essential for the immune response to bacterial challenge, which may contribute to a better understanding of the immune defense system of freshwater mollusks.

## Data Availability Statement

The original contributions presented in the study are included in the article/**Supplementary Material**. Further inquiries can be directed to the corresponding author.

## Author Contributions

FQ, QS, and JL designed the experiments and wrote the manuscript. QS, XZ, YL, XLiu, LR, and ZL conducted the experiments. CG and XLu analyzed the data. ML and XLi modified the manuscript. All authors contributed to the article and approved the submitted version.

## Funding

This research was supported by the Science and Technology Innovation Program of Hunan Province (Grant No. 2020RC3053), the Training Program for Excellent Young Innovators of Changsha (Grant Nos. kq1707015 and kq2106067), the grant from CAS Key Laboratory of Tropical Marine Bio-Resources and Ecology (LMB151007), the Project of Scientific Research of the Hunan Provincial Education Department, China (Grant No. 20A045), and the Talent Introduction Project of Changsha University (Grant No. SF1505).

## Conflict of Interest

The authors declare that the research was conducted in the absence of any commercial or financial relationships that could be construed as a potential conflict of interest.

## Publisher’s Note

All claims expressed in this article are solely those of the authors and do not necessarily represent those of their affiliated organizations, or those of the publisher, the editors and the reviewers. Any product that may be evaluated in this article, or claim that may be made by its manufacturer, is not guaranteed or endorsed by the publisher.
