# Supplementary material for: Molecular Characterization of MyD88 in Anodonta woodiana and Its Involvement in the Innate Immune Response to Bacterial Infection
Source: Front Immunol. 2022 Jun 10;13:925168. doi: 10.3389/fimmu.2022.925168 (PMC9226314; doi:10.3389/fimmu.2022.925168)
Supplement: Supplementary file 1 [file DataSheet_1.docx]

**FIGURE S1.** Nucleotide and deduced amino acid sequences of *Aw*MyD88. The start and stop codons are marked in red. The conserved DEATH domain and TIR domain are indicated by underlined text and gray shading, respectively.


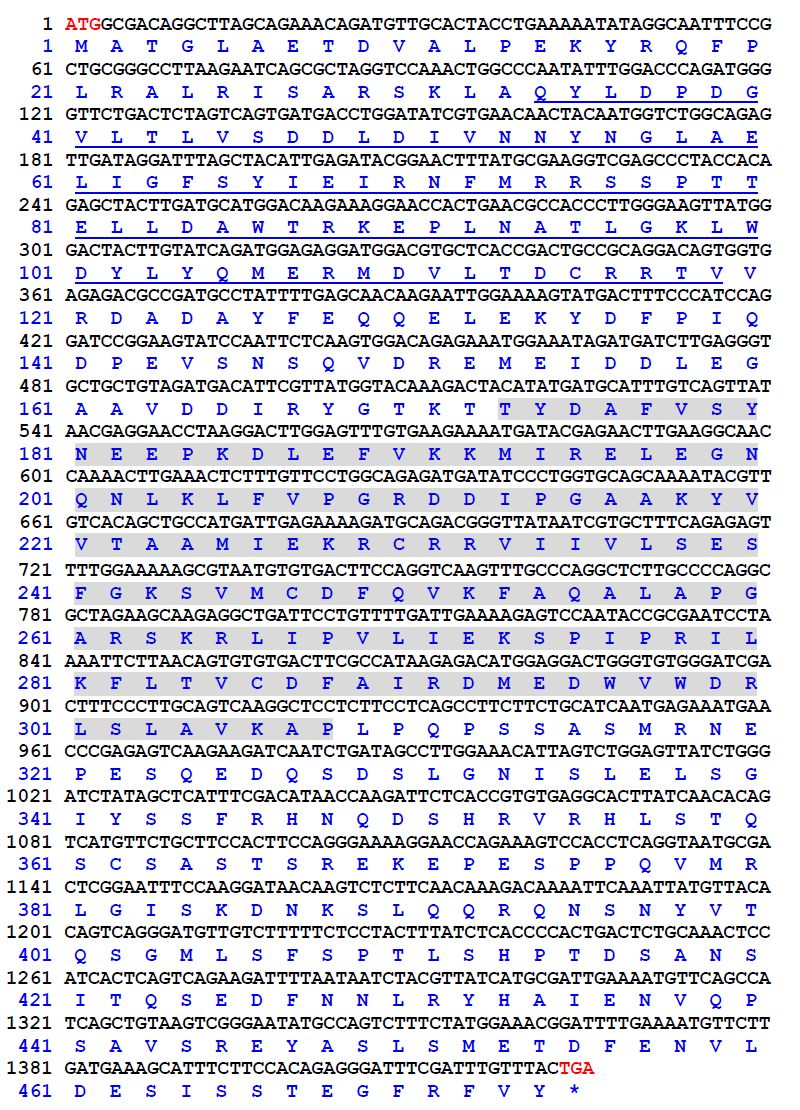


**FIGURE S2.** Multiple sequence alignment of MyD88s from *A. woodiana* and other species. (A) Conserved amino acid residues in these sequences are shown in black. The DEATH and TIR domains are indicated by the red and blue solid-line boxes, respectively. Three typical boxes in the TIR domain are shown as purple dotted-line boxes. (B) The similarities (green) and identities (blue) of MyD88 sequences were analyzed by MatGAT2.02 software using the default settings (BLOSUM50, first gap 12, extending gap 2).

**A**

**
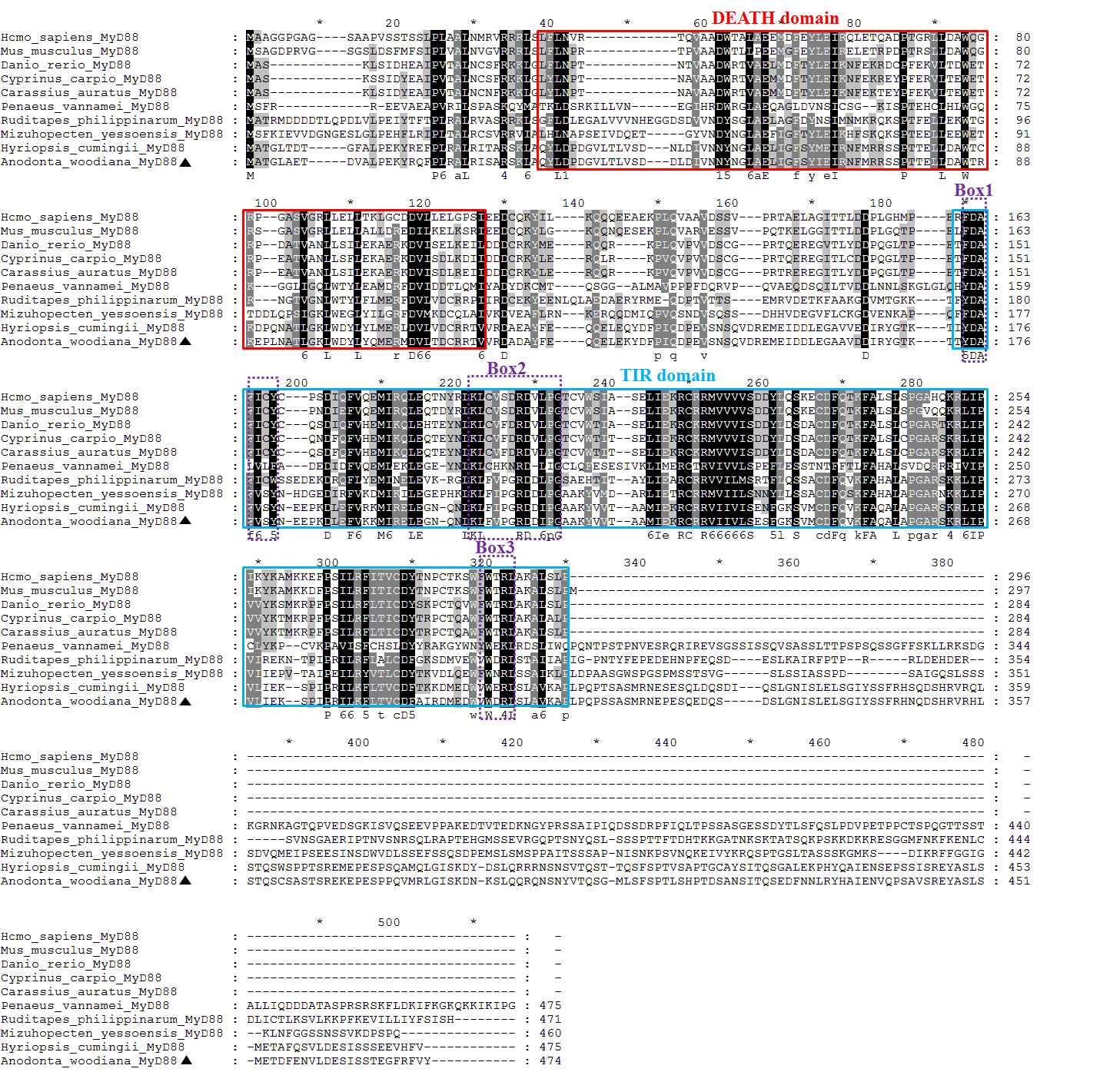
**

**B**

**
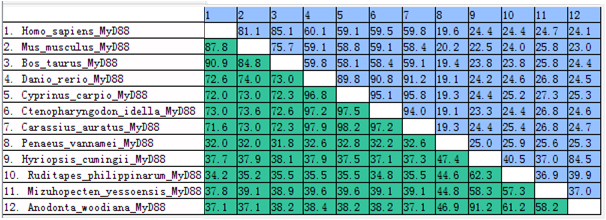
**

**FIGURE S3.** Predicted diagram of innate immunity signaling that *Aw*MyD88 may be involved in *Anodonta woodiana*. Upon *A. hydrophila* stimulation, *Aw*TLRs are activated and then recruit the cytosolic adaptor protein *Aw*MyD88 in *A. woodiana*. Subsequently, *Aw*MyD88 is involved in the production of immune effectors through activation of NF-κB and AP-1 signaling pathways.

**
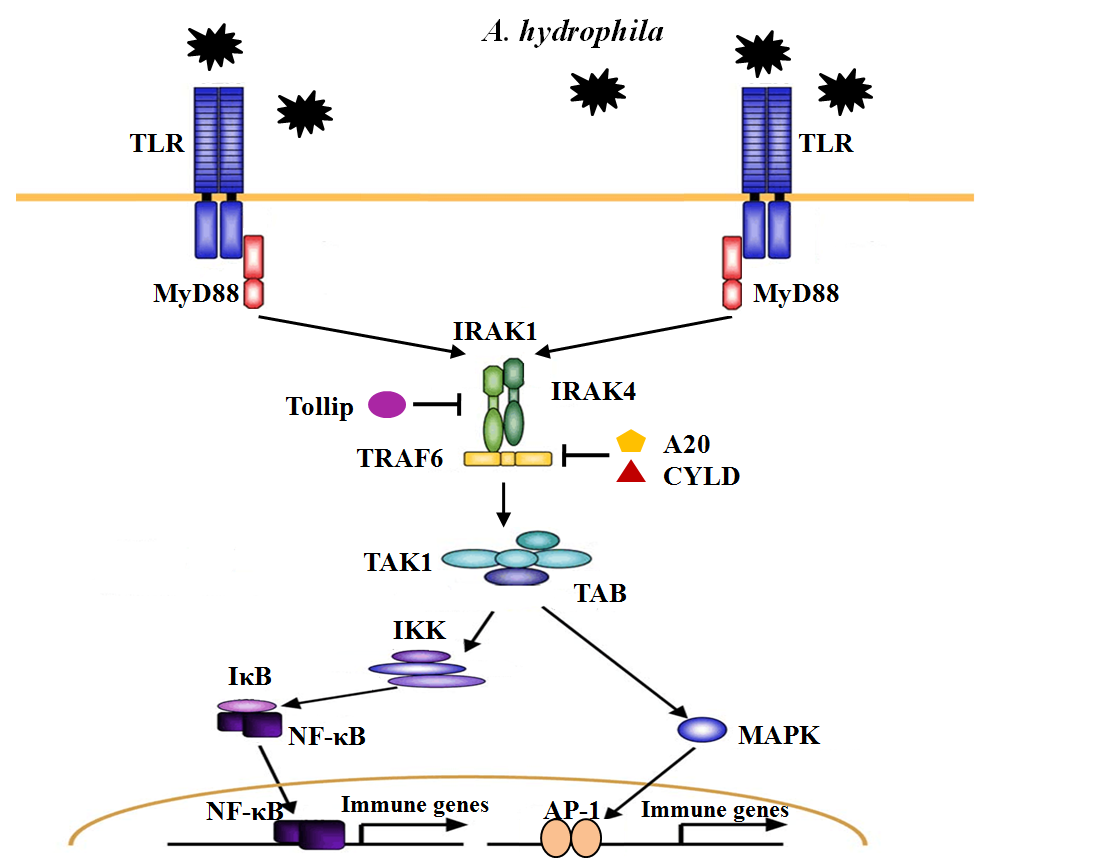
**

**Table S1**

Sequences of designed primers used in this study.

| Primer | Sequence (5’ to 3’ ) | Comment |
| --- | --- | --- |
| *Aw*MyD88-F1 | ATGGCGACAGGCTTAGCAGAA | CDS Cloning |
| *Aw*MyD88-R1 | AAGATAAAAGGTTTCCGATTG |  |
| *Aw*MyD88-F2 | CTCAGGTAATGCGACTCGGAAT | Real-Time PCR |
| *Aw*MyD88-R2 | TCAGTGGGGTGAGATAAAGTAGG |  |
| *Aw*β-actin-F | GTGGCTACTCCTTCACAACC | Real-Time PCR |
| *Aw*β-actin-R | GAAGCTAGGCTGGAACAAGG |  |
| *Aw*TLR-F1 | CTTTTCTGGGGCTGATGTTCC | Real-Time PCR |
| *Aw*TLR-R1 | AGTCTTGCTTGGAAGTGGAGG |  |
| *Aw*TRAF6-F | TGAACAGCAAGGTCAAGAGCAA | Real-Time PCR |
| *Aw*TRAF6-R | AGTTCTGGTCCTTCTTGGGTCC |  |
| *Aw*IRAK1-F | AATGGCTCACTGGAAGATAGACT | Real-Time PCR |
| *Aw*IRAK1-R | GAAGGAACTGAAGACCACACGC |  |
| *Aw*IRAK4-F | TAATAACAGGACTGCCCGCTTT | Real-Time PCR |
| *Aw*IRAK4-R | CATCGTCGGACCAATCTTTCAC |  |
| *Aw*TAK1-F | ATCCAAAGGAGCGTATCACAGT | Real-Time PCR |
| *Aw*TAK1-R | TTGCCTCCCTTAGTTCATCTTT |  |
| *Aw*TAB-F | AGCATTGGTTGACAAAGTGGTT | Real-Time PCR |
| *Aw*TAB-R | CCTCCTGTTGGTGTTCCTATTG |  |
| *Aw*IKK-F | CAAACTGCTGGCGAAAATAGAT | Real-Time PCR |
| *Aw*IKK-R | TTCATTCTTTTTCTGCCATTTT |  |
| *Aw*IκB-F | TTATTGAGAAGTTACCCACGAA | Real-Time PCR |
| *Aw*IκB-R | GCACCTAAGTTGTCCTCCTCCA |  |
| *Aw*NF-κB-F | ACGGAAGAAGGCATCTGAGGTG | Real-Time PCR |
| *Aw*NF-κB-R | CTTGCTTCTCTGCTTGACTGCG |  |
| *Aw*JNK-F | ATTGTTCCCAGATGTGTTATTTCC | Real-Time PCR |
| *Aw*JNK-R | GTTGACTTCGGAATCATCATACCA |  |
| *Aw*p38-F | CAACTTGAAAAATGGGAGAAAA | Real-Time PCR |
| *Aw*p38-R | GAGTGTCAGAGTCAAGGTCCAA |  |
| *Aw*AP-1-F | GAGAAAATGGAGGCAACGCTT | Real-Time PCR |
| *Aw*AP-1-R | TTGTAGAACCGCTGACTGAACC |  |
| *Aw*A20-F | CAAAACAACAGGAGATGGCAAC | Real-Time PCR |
| *Aw*A20-R | CCGCTGGGTCAGTTGATAATGT |  |
| *Aw*Tollip-F | TCTTTGAGACCCCAACAGCCTA | Real-Time PCR |
| *Aw*Tollip-R | CATTCATCTATTGTGCCACCAGT |  |
| *Aw*CYLD-F | CAAATGTCGGAAAGATAAACGG | Real-Time PCR |
| *Aw*CYLD-R | TCTAAAGAGTCTGGTGGGGAAA |  |
| *Aw*TNF-F | TTCTGGAAACAAGGACAGTATC | Real-Time PCR |
| *Aw*TNF-R | ATACTCTCCTTGCTGAAACGAC |  |
| *Aw*Defense-F | ACTACCTACAACCCAGGAGATG | Real-Time PCR |
| *Aw*Defense-R | CTGAAAAGTCCCTACTGGTGAG |  |
| *Aw*LYZ-F | GAAAAATCGGCTGTATGTGGTA | Real-Time PCR |
| *Aw*LYZ-R | TGGCACAAGAGTAATCCAAGGC |  |
| *Aw*AIF-F | ATTTCCTACAGAGATTTTATTCG | Real-Time PCR |
| *Aw*AIF-R | TGTCTTTCACTTCTTCTTTACG |  |
| *Aw*MyD88-F3 | GATAAGAGCCCGGGCGGATCCATGGCGACAGGCTTA | *Aw*MyD88-Flag |
| *Aw*MyD88-R3 | ATCGAATTCCTGCAGAAGCTTTCAGTAAACAAATCG |  |
| *Aw*TLR-F2 | GATAAGAGCCCGGGCGGATCCATGGCTATTCTGGGA | *Aw*TLR-Flag |
| *Aw*TLR-R2 | ATCGAATTCCTGCAGAAGCTTTTAAGTTGTTTTCAG |  |
| *Aw*MyD88-F4  *Aw*MyD88-R4 | CTACCGGACTCAGATCTCGAGATGGCGACAGGCTTA ATGGTGGCGACCGGTGGATCCCGGTAAACAAATCGAA | *Aw*MyD88-GFP |

“F” indicates forward primers and “R” indicates reverse primers.

**Data file 1**

The original data of Figure 3.

| foot | mantle | heart | muscle | gill | hepatopancreas | hemocytes |
| --- | --- | --- | --- | --- | --- | --- |
| 0.596850039 | 2.166611373 | 1.161058 | 3.878343 | 10.450070 | 7.389314 | 14.078680 |
| 0.568582026 | 0.943072376 | 3.106824 | 3.000994 | 5.919357 | 6.043735 | 13.227240 |
| 1.834568024 | 1.29723549 | 0.5232022 | 3.669136 | 4.977566 | 9.616023 | 10.969610 |

**Data file 2**

The original data of Figure 4.

| PBS 0h | PBS 3h | PBS 6h | PBS 12h | PBS 24h |
| --- | --- | --- | --- | --- |
| 0.95322 | 0.779641 | 2.130041 | 1.366875 | 1.249093 |
| 0.387128 | 1.42492 | 2.115328 | 1.06502 | 1.444811 |
| 1.659652 | 1.311194 | 1.043103 | 1.405303 | 1.133576 |

| Ah 0h | Ah 3h | Ah 6h | Ah 12h | Ah 24h |
| --- | --- | --- | --- | --- |
| 1.47517 | 1.71818 | 9.855172 | 8.46131 | 4.379849 |
| 1.28421 | 3.786542 | 13.00398 | 10.4171 | 4.629579 |
| 0.717416 | 4.440989 | 9.195201 | 8.229935 | 3.947341 |

| LPS 0h | LPS 3h | LPS 6h | LPS 12h | LPS 24h |
| --- | --- | --- | --- | --- |
| 0.599105 | 8.69919 | 6.108767 | 3.48433 | 1.141461 |
| 2.56842 | 10.13224 | 5.429732 | 3.319305 | 1.570127 |
| 1.293142 | 4.893549 | 7.840149 | 3.582287 | 2.752764 |

**Data file 3**

The original data of Figure 5.

| PBS 0h | PBS 3h | PBS 6h | PBS 12h | PBS 24h | PBS 48h | PBS 72h |
| --- | --- | --- | --- | --- | --- | --- |
| 0.94927 | 0.739638 | 1.176817 | 1.105647 | 0.226078 | 0.32644 | 0.200947 |
| 1.067984 | 0.635027 | 1.046005 | 1.113337 | 0.413194 | 0.28815 | 0.357221 |
| 0.982746 | 0.576299 | 0.923312 | 1.287784 | 0.196812 | 0.549005 | 0.347453 |

| Ah 0h | Ah 3h | Ah 6h | Ah 12h | Ah 24h | Ah 48h | Ah 72h |
| --- | --- | --- | --- | --- | --- | --- |
| 1.333197 | 0.4553 | 6.254482 | 7.916651 | 3.90383 | 2.703616 | 0.512239 |
| 1.361211 | 0.94927 | 6.565434 | 7.036658 | 3.518329 | 1.796128 | 0.284183 |
| 1.235325 | 0.44285 | 6.341791 | 6.211279 | 3.30555 | 1.7714 | 0.501698 |

| LPS 0h | LPS 3h | LPS 6h | LPS 12h | LPS 24h | LPS 48h | LPS 72h |
| --- | --- | --- | --- | --- | --- | --- |
| 1.351808 | 1.075413 | 2.647976 | 4.975668 | 2.611521 | 1.003395 | 0.464867 |
| 1.226792 | 1.128879 | 2.38649 | 3.958326 | 1.979163 | 1.03878 | 0.38286 |
| 1.067984 | 1.060607 | 2.150825 | 4.126419 | 1.60758 | 0.771047 | 0.385523 |

**Data file 4**

The original data of Figure 6.

| *Aw*TLR | | *Aw*TRAF6 | | *Aw*IRAK1 | | *Aw*IRAK4 | |
| --- | --- | --- | --- | --- | --- | --- | --- |
| PBS | Ah | PBS | Ah | PBS | Ah | PBS | Ah |
| 0.758802 | 5.782907 | 0.56323 | 5.248099 | 0.310313 | 3.486537 | 0.71787 | 3.510788 |
| 1.267332 | 7.421933 | 0.504105 | 5.58592 | 0.457485 | 1.868389 | 1.312025 | 2.754509 |
| 0.973866 | 7.421933 | 0.703097 | 6.642816 | 0.330288 | 3.60949 | 0.830352 | 3.949842 |

| *Aw*TAK1 | | *Aw*TAB | |
| --- | --- | --- | --- |
| PBS | Ah | PBS | Ah |
| 0.540286 | 5.211848 | 1.015222 | 5.10459 |
| 0.877697 | 3.710966 | 0.877697 | 3.736778 |
| 1.267332 | 4.352351 | 1.285024 | 6.596931 |

| *Aw*IKK | | *Aw*IκB | | *Aw*NF-κB | | *Aw*JNK | |
| --- | --- | --- | --- | --- | --- | --- | --- |
| PBS | Ah | PBS | Ah | PBS | Ah | PBS | Ah |
| 0.765598 | 4.838832 | 1.287577 | 3.930352 | 0.556579 | 6.610039 | 1.113158 | 5.009473 |
| 1.278683 | 3.95769 | 2.226316 | 4.18335 | 0.885558 | 4.73925 | 1.113158 | 5.597019 |
| 0.955719 | 5.150308 | 0.942562 | 6.384876 | 0.63054 | 7.183359 | 0.592406 | 5.794398 |

| *Aw*p38 | | *Aw*AP-1 | |
| --- | --- | --- | --- |
| PBS | Ah | PBS | Ah |
| 0.290107 | 3.616662 | 0.36215 | 2.353257 |
| 0.474559 | 3.616662 | 0.600676 | 1.783435 |
| 0.344998 | 5.9573 | 0.335564 | 3.95769 |

| *Aw*A20 | | *Aw*Tollip | | *Aw*CYLD | |
| --- | --- | --- | --- | --- | --- |
| PBS | Ah | PBS | Ah | PBS | Ah |
| 0.877447 | 4.145035 | 1.648763 | 4.963591 | 0.563069 | 6.733629 |
| 1.182126 | 4.504554 | 1.730734 | 5.469406 | 1.110635 | 7.471429 |
| 0.940425 | 6.875117 | 2.481796 | 7.575727 | 0.748142 | 7.62842 |

**Data file 5**

The original data of Figure 7.

| *Aw*MyD88 | | |
| --- | --- | --- |
| PBS | dsEGFP | ds*Aw*MyD88 |
| 3.737463 | 3.253651 | 0.877858 |
| 2.832468 | 2.793472 | 1.134502 |
| 2.679678 | 2.398379 | 0.987642 |

| *Aw*MyD88 | | | *Aw*NF-κB | | |
| --- | --- | --- | --- | --- | --- |
| PBS | Ah | ds*Aw*MyD88+Ah | PBS | Ah | ds*Aw*MyD88+Ah |
| 1.518202 | 6.645435 | 1.0298 | 2.382314 | 19.32456 | 2.269483 |
| 0.679414 | 6.243538 | 1.24174 | 1.476687 | 15.48032 | 2.833065 |
| 0.802383 | 6.20041 | 1.096088 | 1.312542 | 10.35578 | 5.588123 |

| *Aw*AP-1 | | |
| --- | --- | --- |
| PBS | Ah | ds*Aw*MyD88+Ah |
| 1.051438 | 4.414848 | 1.088517 |
| 0.848134 | 4.634339 | 1.615929 |
| 0.703374 | 7.171941 | 1.096088 |

| *Aw*TNF | | | *Aw*LYZ | | |
| --- | --- | --- | --- | --- | --- |
| PBS | Ah | ds*Aw*MyD88+Ah | PBS | Ah | ds*Aw*MyD88+Ah |
| 1.325711 | 6.306179 | 1.927547 | 1.598553 | 7.193861 | 1.620868 |
| 0.756161 | 7.447552 | 0.905487 | 0.874643 | 7.499353 | 2.276419 |
| 0.918127 | 3.045684 | 0.874643 | 1.069374 | 4.913552 | 1.004701 |

| *Aw*Defense | | | *Aw*AIF | | |
| --- | --- | --- | --- | --- | --- |
| PBS | Ah | ds*Aw*MyD88+Ah | PBS | Ah | ds*Aw*MyD88+Ah |
| 0.478558 | 4.812432 | 0.970477 | 1.382008 | 9.169012 | 3.15309 |
| 0.286532 | 5.802868 | 1.107085 | 0.788272 | 10.67946 | 2.921615 |
| 0.640276 | 3.80202 | 1.130347 | 1.262923 | 7.045813 | 2.881393 |

**Data file 6**

The original data of Figure 9.

| pNF-κB Luc | | |
| --- | --- | --- |
| *Aw*MyD88-Flag (0 ng) | *Aw*MyD88-Flag (300 ng) | *Aw*MyD88-Flag (600 ng) |
| 0.916929326 | 5.366090111 | 15.8619798 |
| 1.163014554 | 5.266638366 | 12.49192338 |
| 0.920056123 | 7.078301285 | 9.986904917 |

| pAP-1 Luc | | |
| --- | --- | --- |
| *Aw*MyD88-Flag (0 ng) | *Aw*MyD88-Flag (300 ng) | *Aw*MyD88-Flag (600 ng) |
| 0.842930037 | 2.739783662 | 5.315910817 |
| 1.123962611 | 2.593992188 | 4.664561859 |
| 1.03310735 | 3.422563846 | 4.478957853 |

| pNF-κB Luc | | | |
| --- | --- | --- | --- |
| pCMV-N-Flag | *Aw*MyD88-Flag | *Aw*TLR-Flag | *Aw*MyD88-Flag+*Aw*TLR-Flag |
| 1.34144503 | 6.70338967 | 2.962980673 | 13.6733502 |
| 0.691409507 | 5.44300382 | 3.694591983 | 13.37204821 |
| 0.967145459 | 5.021214756 | 2.746404452 | 18.61947974 |

| pAP-1 Luc | | | |
| --- | --- | --- | --- |
| pCMV-N-Flag | *Aw*MyD88-Flag | *Aw*TLR-Flag | *Aw*MyD88-Flag+*Aw*TLR-Flag |
| 1.212752738 | 2.620080921 | 2.011559227 | 6.490074603 |
| 0.649217355 | 2.898685358 | 2.792718149 | 6.281337625 |
| 1.138029909 | 3.065635991 | 2.979001247 | 7.266142602 |
